# Joint Physical-Psychosocial Frailty and Risks of All-Cause and Cause-Specific Premature Mortality

**DOI:** 10.1007/s11606-024-09335-z

**Published:** 2025-01-22

**Authors:** Jian Zhou, Minghao Kou, Rui Tang, Xuan Wang, Xiang Li, Yoriko Heianza, JoAnn E. Manson, Lu Qi

**Affiliations:** 1 Department of Epidemiology, Tulane University School of Public Health and Tropical Medicine, 1440 Canal Street, Suite 1724, New Orleans, LA 70112 USA; 2 Department of Orthopedics, The Second Xiangya Hospital of Central South University, Changsha, China; 3 Channing Division of Network Medicine, Department of Medicine, Brigham and Women’s Hospital, Harvard Medical School, Boston, MA USA; 4 Department of Epidemiology, Harvard T.H. Chan School of Public Health, Boston, MA USA; 5 Division of Preventive Medicine, Department of Medicine, Brigham and Women’s Hospital, Harvard Medical School, Boston, MA USA; 6 Department of Nutrition, Harvard T.H. Chan School of Public Health, Boston, MA USA

**Keywords:** physical-psychosocial frailty, premature mortality, relative importance, UK Biobank

## Abstract

**Background:**

The importance of integrating physical and psychosocial factors in assessing frailty -health outcomes has been increasingly acknowledged, while the related evidence is lacking. We sought to investigate the associations of joint physical-psychosocial frailty with risk of premature mortality and evaluate the relative importance of individual physical and psychosocial factors.

**Design:**

A total of 381,295 participants with no history of cancer or cardiovascular disease (CVD) were recruited from the UK Biobank cohort. The physical-psychosocial frailty was evaluated based on seven indicators including weight loss, exhaustion, physical activity, walking pace, grip strength, social isolation, and loneliness. The outcomes were premature mortality from all causes, cancer, CVD, and other causes. Cox proportional hazards models were used to assess the associations between the physical-psychosocial frailty and premature mortality.

**Key Results:**

During a median follow-up period of 12.7 years, we recorded 20,328 premature deaths. Each additional increment in the physical-psychosocial frailty index was associated with a 26% (HR 1.26, 95% CI 1.24–1.28), 10% (HR 1.10, 95% CI 1.08–1.12), 30% (HR 1.30, 95% CI 1.26–1.33), and 44% (HR 1.44, 95% CI 1.41–1.47) higher risk of all-cause, cancer, cardiovascular, and other-cause premature mortality, respectively. Compared with participants with the physical-psychosocial frailty index of 0, those with the index ≥ 4 had a 2.67 (95% CI 2.49–2.87)-fold higher risk of all-cause premature mortality. Slow walking pace and social isolation were the top two strongest predictors for all-cause premature mortality. In addition, we found that lower body mass index (BMI), age, smoking status, and dietary quality modified the associations of physical-psychosocial frailty with all-cause premature mortality (*P*-interaction < 0.05).

**Conclusions:**

In this cohort study of UK Biobank participants, joint physical-psychosocial frailty is significantly associated with risks of all-cause and cause-specific premature mortality, highlighting the importance to jointly assess physical and psychosocial factors in determining aging-related health.

**Supplementary Information:**

The online version contains supplementary material available at 10.1007/s11606-024-09335-z.

## INTRODUCTION

Frailty is commonly defined as an aging-related syndrome of physiological decline characterized by apparent vulnerability to adverse health outcomes. With the swift rise in the aging population, the prevalence of frailty is anticipated to grow correspondingly. Previous studies have indicated that individuals living with frailty are susceptible to a range of chronic diseases and premature mortality.^1–4^

Currently, the most widely used definition of frailty was based on the physical phenotypes including weight loss, exhaustion, low physical activity, slow walking pace, and low grip strength.^5^ However, this definition overlooks the psychosocial factors such as social isolation and loneliness, which also contribute to vulnerability to adverse aging-related health outcomes. Social isolation refers to the lack of social contact or support, and loneliness refers to the feeling of being alone or isolated.^6^ Social isolation and loneliness are widespread particularly in the elderly populations.^7^ Emerging evidence has linked social isolation and loneliness with aging related diseases such as CVD and mortality.^8–10^ Notably, these psychosocial factors and physical frailty are significantly correlated and may mutually modulated,^11,12^ and therefore would be considered aging-related risk factors in an integrated manner. However, no study has jointly analyzed these physical and psychological factors simultaneously in assessing frailty and health outcomes.

In this study, we developed a joint physical-psychosocial frailty index comprised of seven indicators: weight loss, exhaustion, low physical activity, slow walking pace, low grip strength, social isolation, and loneliness. We investigated the associations of joint physical-psychosocial frailty with all-cause and cause-specific premature mortality including cancer-, CVD-, and other cause-specific premature mortality. Additionally, we evaluated the relative importance of joint physical-psychosocial frailty indicators in predicting premature mortality, and assessed the interactions between joint physical-psychosocial frailty and other traditional risk factors in relation to the outcomes.

## METHODS

### Study Population

The UK Biobank is a vast biomedical database containing comprehensive genetic and health information from roughly half a million UK residents. Individuals aged between 40 and 69 years were recruited between 2006 and 2010, with biological samples and detailed health data collected. The resource also features a longitudinal study, tracking the health of these individuals over time. The combination of imaging and health data creates a rich resource for studying the links between genetics, lifestyle, and disease. This database is available to researchers worldwide, fostering global collaboration in tackling serious illnesses and advancing our understanding of human health.^13^ The UK Biobank study was approved by the National Health and Social Care Information Management Board and the North West Multicentre Research Ethics Committee (11/NW/0382) and the Institutional Review Board of Tulane University (2018–1872).

### Participant Recruitment

We included 502,411 participants from the UK Biobank in our study. Out of these, 44 participants withdrew from the UK Biobank, and 58,939 participants lacked frailty data; thus, they were excluded from the study. Additionally, we excluded 37,596 participants diagnosed with cancer and 24,537 participants diagnosed with CVD at baseline. As a result, a total of 381,295 participants were enrolled in this study (Supplement Fig. 1).

### Assessment of Joint Physical-Psychosocial Frailty

Joint physical-psychosocial frailty was assessed using seven frailty indicators including weight loss, exhaustion, physical activity, walking pace, grip strength, social isolation, and loneliness (Supplement Table 1). Physical frailty was evaluated by Fried phenotype including five indicators (weight loss, exhaustion, low grip strength, low physical activity, slow walking pace).^2,5,14,15^ The weight loss was evaluated via a question: “Compared with one year ago, has your weight changed?” The response of Yes-lost weight was classified as weight loss and other responses were considered not. The exhaustion was assessed via a question: “Over the past two weeks, how often have you felt tired or had little energy?” The response including more than half the days or nearly every day were considered as exhaustion, while other responses were considered not. The physical activity was assessed via two questions including (1) “In the last 4 weeks did you spend any time doing the following?” and (2) “How many times in the last 4 weeks did you do light DIY?” The responses including no activity and light activity with a frequency of once per week or less were classified as low physical activity, while other responses were considered not. The walking pace was assessed via a question: “How would you describe your usual walking pace?” The response of slow pace was classified as slow walking pace and other responses were considered not. Grip strength was evaluated using a Jamar J00105 hydraulic hand dynamometer. Measurements were taken for both the right and left hands, and the greater value between the two was used for analysis. The low grip strength was defined according to sex and BMI adjusted cutoffs.^5^

Psychosocial frailty was assessed by two indicators including social isolation and loneliness. The social isolation index were calculated by three questions:^16,17^ (1) “Including yourself, how many people are living together in your household?” (2) “How often do you visit friends or family or have them visit you?” (3) “Which of the following (sports club or gym, pub or social club, religious group, adult education class, other group activity) do you attend once a week or more often?” The responses including living alone, having friends and family visit less than once a month, and no participating in social activity at least once per week were defined as high-risk factors, which were coded as index = 1, and those responses with low-risk factors were coded as index = 0. Social isolation status was classified as isolation (isolation index ≥ 2) and non-isolation (isolation index < 2).^16–18^ The loneliness index was assessed via two questions: (1) “Do you often feel lonely?” (2) “How often are you able to confide in someone close to you?” The responses including feeling lonely and once every few months/never or almost never being able to confide were defined as high-risk factors, which were coded as index = 1, and those responses with low-risk factors were coded as index = 0. The loneliness status was classified as loneliness (loneliness index = 2) and non-loneliness (loneliness scale < 2).^16–18^ The total index of joint physical-psychosocial frailty ranges from 0 to 7.

### Outcomes

The main outcomes of this research included all-cause premature mortality, as well as premature mortality from cancer, CVD, and other. Information on the cause and date of death were obtained by linking to the Death Registry of the National Health Service (NHS) Information Centre for England and Wales participants, and to the Death Registry of the Scottish NHS Central Registry for those from Scotland. More information about these death registries can be found at http://content.digital.nhs.uk/services. The premature deaths were defined as death occurring before the age of 80.^19^ The final date for the follow-up was established as the earlier of the two dates: either the date of death or the censoring date (November 27, 2021). Mortality data was classified according to the International Classification of Diseases, 10th Revision (ICD 10). In this analysis, we examined all-cause premature mortality, cancer premature mortality (codes C00 to C97), CVD premature mortality (codes I00 to I99), and other premature mortality.^20,21^

### Evaluation of Other Variables

Age, sex, ethnic background, Townsend deprivation index, education years, smoking status, and alcohol intake frequency were self-reported. BMI was calculated by dividing weight in kilograms by the square of height in meters (kg/m^2^). A Healthy Diet Score was generated based on intake of vegetables, fruits, fish, processed meats, and unprocessed red meats (Supplement Table 2). The methodology for this scoring system has been elaborated in our previous research.^22,23^ We classified hypertension as either having a systolic blood pressure of 140 mmHg or more, a diastolic blood pressure of 90 mmHg or more, or if the individual was taking antihypertensive medication. High cholesterol condition was identified either by a self-reported history of high cholesterol levels or using cholesterol-lowering drugs. Diabetes was characterized by a self-reported history of the condition or the use of insulin. Comprehensive information about all variables incorporated in the study is accessible on the UK Biobank website (www.ukbiobank.ac.uk).

### Statistical Analysis

The presentation of continuous variables was indicated as mean ± standard deviation (SD), with categorical variables displayed as counts and percentages. We used ANOVA tests or *χ*^2^ tests to examine participant characteristics. The association between joint physical-psychosocial frailty and the probability of mortality from all causes, cancer, CVD, and other causes was analyzed using Cox proportional hazard regression models. The proportionality of hazards was authenticated via Schoenfeld residuals and Kaplan–Meier methods, with all analyses adhering to predefined conditions. The basic model was adjusted for age (years) and sex (male or female) and the multivariable model was adjusted for age (years) and sex (male or female), ethnic background (white or others), Townsend deprivation index (continuous), education years (continuous), BMI (< 25, 25– < 30, or ≥ 30 kg/m^2^), smoking status (never, previous or current smoking), alcohol intake (< 3 or ≥ 3 times/week), healthy diet score (< 3 or ≥ 3), hypertension (yes or no), high cholesterol (yes or no), and diabetes (yes or no). Missing values for categorical predictors and continuous variables were resolved with an indicator category for missing data and mean values, respectively. Supplement Table 3 provides a thorough account of the count and percentage of participants with missing covariates.

We evaluated the relative importance of seven physical-psychosocial frailty indicators utilizing the *coxphERR* package from R version 4.1.2 (www.r-project.org)^24^ *R*^2^ was generated by developed applications for the multivariable model, is confined within a range from 0 to 1. Risk factors that present a significant and explicit *R*^2^ measure are recognized as relevant.

Additionally, we performed a series of stratified analyses according to age (≥ 60 vs < 60 years), sex (male vs female), ethnic background (white vs others), Townsend Deprivation Index (≥ median vs < median), education years (< 10 vs ≥ 10 years), BMI (< 25, 25– < 30 or ≥ 30 kg/m^2^), smoking status (never vs previous/current smoking), alcohol intake (< 3 vs ≥ 3 times/week), healthy diet score (< 3 vs ≥ 3), hypertension (yes or no), high cholesterol (yes or no), and diabetes (yes or no). We used the same Cox model by adding interaction terms.

### Sensitivity Analyses

To demonstrate the robustness of the results, we conducted three sensitivity analyses. Firstly, we removed those participants who died within the first 2 years of follow-up period. Second, we deleted the participants with missing data for covariates. Third, we imputed the missing data for all covariates using multiple imputation. Our statistical examinations were accomplished using SAS version 9.4 (SAS Institute, Cary, NC) and R version 4.1.2 (www.r-project.org), and a two-sided *P* value < 0.05 was considered statistically significant.

## RESULTS

### Baseline Characteristics

Table 1 shows the baseline characteristics of the participants included in the study. The average age of the study population was 55.8 years old, of which 55.1% (210,117 participants) were identified as female. It was observed that individuals with higher joint physical-psychosocial frailty index were predominantly older females of non-white ethnicity. Additionally, they were more likely to be current smokers, with a lower socioeconomic background, and fewer years of education. These individuals also exhibited a higher BMI, consumed alcohol less frequently, had lower scores for healthy diet, and reported hypertension, high cholesterol, and diabetes.

**Table 1:** Baseline Characteristics for Included Participants

Characteristics	Total (*n* = 381295)	Physical-psychosocial frailty index					*P*-value
		**0 (*n* = 207,093)**	**1 (*n* = 114,979)**	**2 (*n* = 39,870)**	**3 (*n* = 13,401)**	** ≥ 4 (*n* = 5952)**	
Age, years, mean (SD)	55.8 (8.1)	55.7 (8.1)	55.9 (8.1)	56.1 (8)	56.3 (7.8)	56.3 (7.7)	< 0.001
Female, *n* (%)	210,117 (55.1)	109,405 (52.8)	64,834 (56.4)	23,829 (59.8)	8326 (62.1)	3723 (62.6)	< 0.001
Ethnic background, *n* (%)							< 0.001
Asian	13,157 (3.5)	6848 (3.3)	4083 (3.6)	1459 (3.7)	542 (4.0)	225 (3.8)	
Black	1840 (0.5)	688 (0.3)	628 (0.6)	322 (0.8)	153 (1.1)	49 (0.8)	
Chinese	1024 (0.3)	440 (0.2)	334 (0.3)	173 (0.4)	57 (0.4)	20 (0.3)	
Mixed	13,304 (3.5)	6054 (2.9)	4334 (3.8)	1890 (4.7)	708 (5.3)	318 (5.3)	
White	348,058 (91.3)	191,563 (92.5)	104,322 (90.7)	35,348 (88.7)	11,651 (86.9)	5174 (86.9)	
Other*	2899 (0.8)	1028 (0.5)	966 (0.8)	538 (1.4)	234 (1.8)	133 (2.2)	
Townsend deprivation index, mean (SD)	− 1.4 (3)	− 1.8 (2.8)	− 1.3 (3)	− 0.6 (3.3)	0.1 (3.5)	0.9 (3.6)	< 0.001
Education, years, mean (SD)	15.2 (5)	15.7 (4.8)	15 (5)	14.2 (5.2)	13.4 (5.3)	12.5 (5.3)	< 0.001
BMI, kg/m, *n* (%)							< 0.001
< 25	130,350 (34.2)	80,986 (39.1)	35,433 (30.8)	9981 (25)	2820 (21)	1130 (19)	
25– < 30	162,437 (42.6)	90,888 (43.9)	49,724 (43.3)	15,527 (38.9)	4535 (33.8)	1763 (29.6)	
≥ 30	88,508 (23.2)	35,219 (17)	29,822 (25.9)	14,362 (36)	6046 (45.1)	3059 (51.4)	
Smoking status, *n* (%)							< 0.001
Never	213,436 (56)	120,046 (58)	63,235 (55)	21,012 (52.7)	6502 (48.5)	2641 (44.4)	
Previous	128,099 (33.6)	69,556 (33.6)	39,247 (34.1)	13,115 (32.9)	4355 (32.5)	1826 (30.7)	
Current	38,818 (10.2)	17,076 (8.3)	12,222 (10.6)	5588 (14)	2478 (18.5)	1454 (24.4)	
Alcohol intake, times/week, *n* (%)							< 0.001
< 3	209,949 (55.1)	101,864 (49.2)	66,830 (58.1)	26,681 (66.9)	9877 (73.7)	4697 (78.9)	
≥ 3	171,191 (44.9)	105,189 (50.8)	48,090 (41.8)	13,163 (33)	3505 (26.2)	1244 (20.9)	
Healthy diet score, *n* (%)							< 0.001
< 3	123,236 (32.3)	64,712 (31.3)	36,765 (32)	14,013 (35.2)	5244 (39.1)	2502 (42)	
≥ 3	247,630 (64.9)	138,302 (66.8)	74,865 (65.1)	24,149 (60.6)	7335 (54.7)	2979 (50.1)	
Hypertension, *n* (%)	213,745 (56.1)	112,609 (54.4)	65,025 (56.6)	23,714 (59.5)	8426 (62.9)	3971 (66.7)	< 0.001
High cholesterol, *n* (%)	1896 (0.5)	812 (0.4)	566 (0.5)	283 (0.7)	140 (1)	95 (1.6)	< 0.001
Diabetes, *n* (%)	16,042 (4.2)	4940 (2.4)	5398 (4.7)	3214 (8.1)	1526 (11.4)	964 (16.2)	< 0.001

*UK Biobank did not define “other” racial and ethnic groups

### Association Between Joint Physical-Psychosocial Frailty and Premature Mortality

The median follow-up time was 12.7 years. Throughout this period, a total of 20,328 premature mortality were recorded, encompassing 10,438 due to cancer, 3850 due to CVD, and 6040 attributed to other causes. Supplement Fig. 2 presents the cumulative hazard curves of premature mortality from all causes, cancer, CVD, and other causes. Age- and sex-adjusted results from basic model indicated significant associations between joint physical-psychosocial frailty and the risk of premature mortality (Table 2). Multivariable model showed that each additional increment in the joint physical-psychosocial frailty index increased the risk of premature mortality by 26% (HR 1.26, 95% CI 1.24–1.28), cancer mortality by 10% (HR 1.10, 95% CI 1.08–1.12), cardiovascular mortality by 30% (HR 1.30, 95% CI 1.26–1.33), and other causes by 44% (HR 1.44, 95% CI 1.41–1.47) (Table 2).

**Table 2:** Hazard Ratios and 95% Confidence Intervals for Association of Physical-psychosocial Frailty with Outcome of Premature Mortality

Outcomes	Physical-psychosocial frailty index					Per 1 index increase	*P*-trend
	**0**	**1**	**2**	**3**	** ≥ 4**		
All-cause premature mortality							
Event, *n* (%)	8650 (4.18)	6347 (5.52)	2987 (7.49)	1446 (10.79)	898 (15.09)		
Basic model	1.00 (Reference)	1.36 (1.32–1.41)	1.90 (1.82–1.98)	2.83 (2.67–2.99)	4.14 (3.87–4.44)	1.39 (1.37–1.41)	< 0.001
Multivariable model	1.00 (Reference)	1.26 (1.21–1.30)	1.57 (1.50–1.64)	2.07 (1.95–2.19)	2.67 (2.49–2.87)	1.26 (1.24–1.27)	< 0.001
Cancer premature mortality							
Event, *n* (%)	4966 (2.40)	3344 (2.91)	1322 (3.32)	527 (3.93)	279 (4.69)		
Basic model	1.00 (Reference)	1.23 (1.17–1.28)	1.40 (1.32–1.49)	1.67 (1.53–1.83)	2.02 (1.79–2.28)	1.18 (1.16–1.20)	< 0.001
Multivariable model	1.00 (Reference)	1.15 (1.10–1.20)	1.21 (1.14–1.29)	1.31 (1.20–1.44)	1.45 (1.28–1.64)	1.10 (1.07–1.12)	< 0.001
CVD premature mortality							
Event, *n* (%)	1493 (0.72)	1192 (1.04)	651 (1.63)	321 (2.40)	193 (3.24)		
Basic model	1.00 (Reference)	1.49 (1.38–1.61)	2.43 (2.21–2.66)	3.64 (3.22–4.11)	5.03 (4.33–5.84)	1.49 (1.45–1.53)	< 0.001
Multivariable model	1.00 (Reference)	1.32 (1.22–1.43)	1.84 (1.67–2.02)	2.35 (2.07–2.67)	2.76 (2.36–3.24)	1.30 (1.26–1.33)	< 0.001
Other premature mortality							
Event, *n* (%)	2191 (1.06)	1811 (1.58)	1014 (2.54)	598 (4.46)	426 (7.16)		
Basic model	1.00 (Reference)	1.53 (1.44–1.63)	2.54 (2.36–2.74)	4.55 (4.15–4.98)	7.58 (6.83–8.41)	1.61 (1.58–1.64)	< 0.001
Multivariable model	1.00 (Reference)	1.41 (1.32–1.50)	2.07 (1.92–2.24)	3.25 (2.95–3.57)	4.68 (4.18–5.23)	1.44 (1.41–1.47)	< 0.001

Basic model: adjusted for age and sex

Multivariable model: adjusted for age, sex, ethnic background, Townsend deprivation index, education years, body mass index, smoking status, alcohol intake, healthy diet score, hypertension, high cholesterol, and diabetes

Supplement Fig. 3 shows positive linear associations of the joint physical-psychosocial frailty with the incidence of all-cause premature mortality (*P*-linearity < 0.001), cancer premature mortality (*P*-linearity < 0.001), CVD premature mortality (*P*-linearity < 0.001), and other premature mortalities (*P*-linearity < 0.001).

### Joint Physical-Psychosocial Frailty Indicators and Premature Mortality

**Figure 1:**
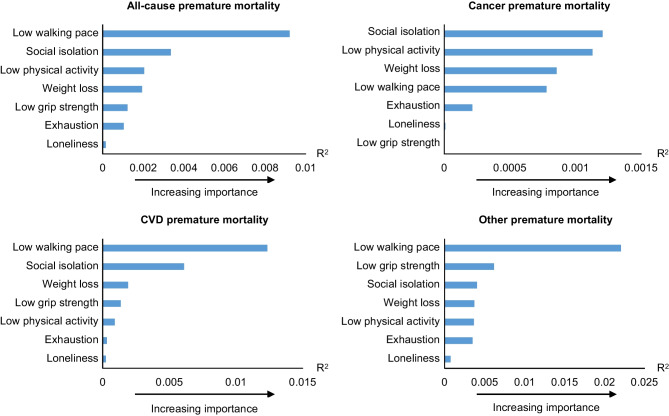
Relative importance of physical-psychosocial frailty indicators. *R*^2^ was generated by developed applications for the multivariable model adjusted for age, sex, ethnic background, Townsend deprivation index, education years, body mass index, smoking status, alcohol intake, healthy diet score, hypertension, high cholesterol, and diabetes. Social isolation, loneliness, weight loss, exhaustion, physical activity, walking pace, and grip strength were mutually adjusted for each other.

The relative importance of joint physical-psychosocial frailty indicators in relation to all-cause and cause-specific premature mortality was assessed by *R*^2^ generated by developed applications for the multivariable model. Slow walking pace and social isolation were the top two strongest predictors for all-cause premature mortality. Slow walking pace ranked first in relative strength for predicting risk of all-cause premature mortality, CVD premature mortality, and other premature mortality. Social isolation ranked second, first, second, and third in relative strength for predicting risk of all-cause premature mortality, cancer premature mortality, CVD premature mortality, and other premature mortality, respectively (Fig. 1).

Additionally, the association of individual joint physical-psychosocial frailty indicators with risk of premature mortality was analyzed via multivariable model. We observed that the results were similar to the relative importance analyses. Slow walking pace showed the strongest association for all-cause premature mortality, CVD premature mortality, and other premature mortality. Social isolation indicated the strongest association for cancer premature mortality (Supplement Fig. 4).

### Stratified Analysis

The results from stratified analyses indicated that the association of joint physical-psychosocial frailty with all-cause premature mortality was stronger among participants who were younger than 60 years old (*P*-interaction = 0.001), male (*P*-interaction = 0.005), previous or current smokers (*P*-interaction < 0.001), with higher alcohol intake frequency (*P*-interaction < 0.001), with lower healthy diet score (*P*-interaction = 0.04), and without high cholesterol (*P*-interaction = 0.013) (Table 3). We also performed stratified analysis for associations of joint physical-psychosocial frailty with cancer, CVD, and other premature mortality (Supplement Tables 4–6). We found that association of joint physical-psychosocial frailty with cancer premature mortality was stronger among previous or current smokers (*P*-interaction < 0.001). The association of joint physical-psychosocial frailty with CVD premature mortality was stronger among younger than 60 years old (*P*-interaction = 0.017) and with lower healthy diet score (*P*-interaction < 0.001). Additionally, we analyzed the interactions between the frailty index components and other covariates (Supplement Table 7).

**Table 3:** Association of Physical-psychosocial Frailty with Risk of Premature Mortality Stratified by Potential Risk Factors via Multivariable Model

Subgroup	Physical-psychosocial frailty index					*P-*trend	*P*-interaction
	**0**	**1**	**2**	**3**	** ≥ 4**		
Age (years)							0.001
< 60	1.00 (reference)	1.25 (1.18–1.32)	1.62 (1.50–1.74)	2.12 (1.92–2.34)	2.96 (2.64–3.32)	< 0.001	
≥ 60	1.00 (reference)	1.27 (1.22–1.32)	1.56 (1.48–1.65)	2.07 (1.93–2.23)	2.53 (2.31–2.78)	< 0.001	
Sex							0.005
Female	1.00 (reference)	1.21 (1.15–1.28)	1.47 (1.37–1.57)	1.95 (1.79–2.13)	2.63 (2.37–2.92)	< 0.001	
Male	1.00 (reference)	1.28 (1.23–1.34)	1.64 (1.55–1.74)	2.17 (2.00–2.34)	2.71 (2.45–2.99)	< 0.001	
Ethnic background							0.076
Non-White	1.00 (reference)	1.28 (1.13–1.46)	1.62 (1.39–1.89)	1.75 (1.42–2.16)	2.50 (1.96–3.18)	< 0.001	
White	1.00 (reference)	1.25 (1.21–1.30)	1.56 (1.49–1.63)	2.11 (1.98–2.24)	2.69 (2.50–2.91)	< 0.001	
Townsend deprivation index							0.396
< Median	1.00 (reference)	1.21 (1.16–1.27)	1.57 (1.47–1.68)	2.07 (1.87–2.30)	2.59 (2.22–3.02)	< 0.001	
≥ Median	1.00 (reference)	1.31 (1.25–1.37)	1.62 (1.53–1.72)	2.16 (2.01–2.32)	2.87 (2.64–3.11)	< 0.001	
Education (years)							0.109
< 10	1.00 (reference)	1.25 (1.17–1.34)	1.52 (1.40–1.65)	2.07 (1.88–2.27)	2.59 (2.31–2.90)	< 0.001	
≥ 10	1.00 (reference)	1.26 (1.21–1.31)	1.59 (1.51–1.67)	2.07 (1.92–2.23)	2.72 (2.47–3.00)	< 0.001	
Smoking status							< 0.001
Never	1.00 (reference)	1.21 (1.15–1.28)	1.50 (1.40–1.61)	2.01 (1.82–2.22)	2.61 (2.29–2.97)	< 0.001	
Previous/current	1.00 (reference)	1.31 (1.26–1.37)	1.69 (1.60–1.79)	2.28 (2.12–2.45)	2.98 (2.73–3.26)	< 0.001	
Alcohol intake (times/week)							< 0.001
< 3	1.00 (reference)	1.24 (1.19–1.30)	1.51 (1.43–1.60)	1.96 (1.83–2.11)	2.50 (2.29–2.73)	< 0.001	
≥ 3	1.00 (reference)	1.26 (1.20–1.32)	1.63 (1.52–1.74)	2.29 (2.07–2.53)	3.11 (2.73–3.55)	< 0.001	
Healthy diet score							0.040
< 3	1.00 (reference)	1.26 (1.20–1.34)	1.60 (1.49–1.72)	2.20 (2.01–2.41)	2.76 (2.47–3.07)	< 0.001	
≥ 3	1.00 (reference)	1.25 (1.20–1.30)	1.55 (1.47–1.64)	1.92 (1.77–2.09)	2.54 (2.29–2.83)	< 0.001	
High cholesterol							0.013
No	1.00 (reference)	1.23 (1.18–1.28)	1.59 (1.51–1.67)	2.09 (1.95–2.24)	2.76 (2.53–3.02)	< 0.001	
Yes	1.00 (reference)	1.33 (1.24–1.42)	1.49 (1.36–1.62)	1.97 (1.77–2.20)	2.47 (2.17–2.80)	< 0.001	
Diabetes							0.402
No	1.00 (reference)	1.26 (1.21–1.30)	1.58 (1.51–1.65)	2.05 (1.92–2.18)	2.68 (2.47–2.91)	< 0.001	
Yes	1.00 (reference)	1.22 (1.08–1.37)	1.44 (1.26–1.64)	2.09 (1.80–2.44)	2.56 (2.17–3.03)	< 0.001	

Multivariable model: adjusted for age, sex, ethnic background, Townsend deprivation index, education years, body mass index, smoking status, alcohol intake, healthy diet score, hypertension, high cholesterol, and diabetes

**Figure 2:**
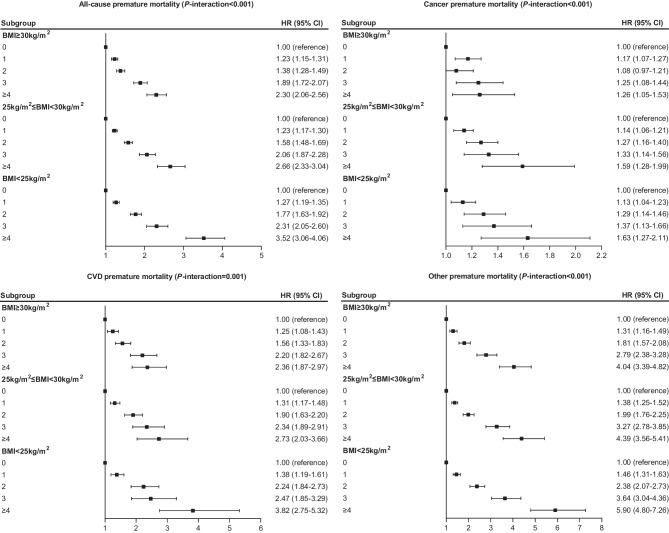
The subgroup analyses of physical-psychosocial frailty with risk of premature mortality by body mass index via multivariable model adjusted for age, sex, ethnic background, Townsend deprivation index, education years, body mass index, smoking status, alcohol intake, healthy diet score, hypertension, high cholesterol, and diabetes.

Additionally, we found that the associations of joint physical-psychosocial frailty with all-cause premature mortality, cancer premature mortality, CVD premature mortality, and other premature mortality were significantly modified by BMI (*P*-interaction < 0.001) (Fig. 2). The association between joint physical-psychosocial frailty and premature mortality was weaker among participants with BMI ≥ 30 kg/m^2^ than other groups with lower BMI level. Then we performed BMI stratified analyses for joint physical-psychosocial frailty indicators. We observed that social isolation, weight loss, exhaustion, low physical activity, slow walking pace, and low grip strength showed similar interaction patterns with BMI on risk of all-cause premature mortality (Supplement Fig. 5).

### Sensitivity Analyses

We performed several sensitivity analyses to affirm the robustness of our results. After excluding the participants who experienced premature mortality within the initial 2 years of the follow-up, the results persisted unaltered (Supplementary Table 8). Upon the removal of participants with absent covariates, the results did not change significantly (Supplementary Table 9). Additionally, we imputed data for all covariate with missing values using multiple imputation, which did not noticeably alter the results (Supplementary Table 10).

## DISCUSSION

In this prospective study involving 381,295 participants, we found that increase of each additional unit of joint physical-psychosocial frailty index was related to a 26%, 10%, 30%, and 44% elevated risk for premature mortality from all causes, cancer, CVD, and other causes, respectively. Compared with participants with the joint physical-psychosocial frailty index of 0, those with the index ≥ 4 had a 2.67 (2.49–2.87)-fold higher risk of all-cause premature mortality. Slow walking pace and social isolation were the top two strongest predictors for all-cause premature mortality. Additionally, we identified that BMI, smoking, alcohol consumption, and healthy diet score significantly modified the associations of joint physical-psychosocial frailty with premature mortality.

To our knowledge, this is the first study analyzing the joint association of psychosocial frailty and physical frailty with premature mortality. Currently, the most widely used definition of frailty is based on the physical phenotypes including weight loss, exhaustion, low physical activity, slow walking pace, and low grip strength. However, such physical frailty has major limitations in defining the overall aging-related physiological declination and vulnerability to adverse health outcomes. Growing evidence has shown that aging is not only related to decline in physical function, but also psychosocial wellbeing and connections.^25–27^ Social isolation and loneliness have been associated with aging related diseases including CVD,^28,29^ cancer,^30^ respiratory disease,^31^ type 2 diabetes,^32^ dementia,^33^ and infection^17^ and mortality.^9,34^ Moreover, elderly individuals with loneliness were at increased risk of physical frailty,^35^ and physical frailty was also related to social isolation^11^ and loneliness,^12^ indicating that psychosocial factors and physical frailty were closely related to each other. Additionally, physically frail individuals with social isolation or loneliness may experience higher risk of mortality compared to physically frail participants without social isolation or loneliness,^36^ suggesting that social isolation, loneliness, and physical frailty may increase the risk of premature mortality through different pathways and they may potentially interact with each other. Emerging evidence suggests that physical frailty, social isolation, and loneliness independently impact health, but their combination may amplify vulnerability through complex mechanisms. For instance, social isolation and loneliness are associated with chronic stress and dysregulation of the hypothalamic–pituitary–adrenal (HPA) axis, leading to elevated cortisol levels, systemic inflammation, and impaired immune function.^37,38^ These physiological changes may exacerbate physical frailty by accelerating muscle loss, reducing mobility, and impairing recovery from illness. Conversely, physical frailty can limit social participation and increase isolation, perpetuating a feedback loop that worsens psychosocial well-being.^5,39^ Behavioral factors further mediate these associations. Socially isolated individuals may lack encouragement or resources to maintain physical activity, adhere to healthy diets, or seek medical care.^40,41^ These behaviors can exacerbate physical decline and compound mortality risk. Additionally, loneliness and physical frailty have both been linked to cognitive decline, which may independently and synergistically increase mortality risk through reduced self-care and decision-making capacity.^42^ Importantly, the pathways linking physical and psychosocial frailty to mortality may differ across specific causes. For example, social isolation and loneliness are strongly linked to cardiovascular mortality due to their association with hypertension, dyslipidemia, and systemic inflammation.^28^ Therefore, it is essential to address psychosocial frailty and physical frailty jointly to comprehensively evaluate the aging related vulnerability to adverse health outcomes.

Additionally, our study found that slow walking pace and social isolation were the top two strongest predictors for all-cause premature mortality among seven joint physical-psychosocial frailty indicators. A previous study has indicated that the social isolation–related risk of premature mortality was comparable with well-established risk factors including physical activity, obesity, and injury.^41^ Similarly, another study presented that the predictive power of social isolation in relation to mortality was similar to that of widely recognized clinical risk factors including smoking, obesity, and high cholesterol.^41^ Although the predictive effect for premature mortality of loneliness was relative weaker than other physical-psychosocial indicators, we observed that it was significantly associated with all-cause premature mortality, which was consistent with the result of a previous study.^9^ Additionally, a meta-analytic review with 70 studies indicated that loneliness-related risk of premature mortality was comparable with traditional risk factors including physical activity and obesity.^41^ These results lend support to the need to consider social isolation and loneliness as significant predictors for premature mortality.

Intriguingly, we found that the association of joint physical-psychosocial frailty with premature mortality was weaker among participants with higher BMI than those with higher BMI. In line with our findings, a cohort study indicated that being overweight was related to lower risk of mortality in frail participants.^43^ Body weight is a composite of various elements including muscle mass, fat mass, bone mineral mass, and body water, and increase in any of these components may lead to an elevation in BMI. People with substantial skeletal muscle mass and strength often experience a superior quality of life and a lower mortality rate,^44,45^ and a certain degree of BMI increase can act as a protective mortality factor in the elderly.^46^ Conversely, sarcopenia, characterized by muscle degradation, contributes to increased rates of infection, prolonged hospital stays, immobilization, and higher mortality.^47,48^ In the elderly, unintentional weight reduction can lead to malnutrition and weakened immunity, impacting negatively on health outcomes such as increasing the risk of infections, pressure ulcers, morbidity, and mortality.^49,50^ Additionally, we observed that the relationship of joint physical-psychosocial frailty with all-cause premature mortality was stronger among participants who were younger male, with unhealthy lifestyle and without high cholesterol. In line with our study, previous studies indicated that the associations between high frailty index and all-cause mortality attenuated with age^51–53^ and the risk of all-cause mortality was stronger in male than that in female.^2^ While elevated levels of serum cholesterol may contribute to an increased incidence of coronary heart disease, they do not seem to influence the hospital outcomes.^54^ However, the in-depth mechanism remains to be explored.

The generalizability of the findings to other populations may be limited. To enhance the generalizability of our findings, we propose that future studies validate the associations identified in our research within more diverse populations. For example, populations in low- and middle-income countries (LMICs) may exhibit distinct patterns of frailty due to differences in healthcare access, dietary habits, and social support systems. Similarly, ethnic and cultural factors may influence psychosocial indicators, such as the perception of loneliness or social isolation, which could affect the relative contributions of these components to overall frailty.

We suggest the following: (1) Validation of the physical-psychosocial frailty index in cohorts with broader ethnic, geographic, and socioeconomic diversity, such as those from Asia, Africa, and Latin America. (2) Longitudinal studies to assess how the relative importance of physical and psychosocial components evolves over time in different populations. (3) Development of culturally sensitive adaptations of the frailty index that incorporate region-specific health behaviors and social norms. (4) Examination of the index’s utility in predicting outcomes other than mortality, such as morbidity or healthcare utilization, to provide a more holistic understanding of frailty’s impact.

The current study’s significant strengths include the development of novel joint physical-psychosocial frailty for comprehensively assessing aging related vulnerability, a substantial sample size and extensive details on covariates. However, there are certain limitations, including (1) the frailty index’s evaluation relied on self-reported responses, making misclassification and recall biases unavoidable. (2) Despite our adjustments for potential confounding variables, residual confounders cannot be entirely discounted. (3) The present study does not establish a definitive cause-effect relationship, as it is observational in nature. While we adjusted for a wide range of potential confounders to minimize bias, the possibility of residual confounding or reverse causality, particularly in the psychosocial aspects of frailty, cannot be entirely ruled out. Future longitudinal studies with repeated measures are needed to further clarify the directionality of these associations. (4) The participants in our study were predominantly from the UK, which may limit the generalizability of our conclusions to populations in other regions with different ethnic, cultural, or socioeconomic characteristics. (5) Unmeasured or unknown confounders may still influence the study outcomes, emphasizing the need for future studies, including experimental or longitudinal designs, to establish causal relationships.

### Future Research Directions

Our findings highlight the need for further research to develop targeted interventions addressing both physical and psychosocial frailty. For example, group-based exercise programs could not only improve physical function but also foster social connections, addressing loneliness and social isolation simultaneously. Integrating physical rehabilitation with mental health support might provide a comprehensive approach to reduce frailty and its associated risks. Additionally, digital technologies, such as virtual social platforms or telemedicine, hold potential for mitigating psychosocial frailty components, particularly social isolation, in populations with limited access to in-person interventions. Investigating these innovative strategies and evaluating their effectiveness in diverse populations could further enhance our understanding of frailty and inform holistic preventive approaches.

## CONCLUSION

The present study showed that joint physical-psychosocial frailty was significantly related to all-cause and cause-specific premature mortality. Our findings highlight the importance of considering psychosocial factors in assessment of aging related frailty, in conjunction with physical factors in relation to premature mortality.

## Supplementary Information

Below is the link to the electronic supplementary material.

Supplementary file1 (DOCX 5.02 MB)

## Data Availability

This study has been conducted using the UK Biobank Resource, approved project number 29256. The UK Biobank will make the source data available to all bona fide researchers for all types of health-related research that is in the public interest, without preferential or exclusive access for any persons. All researchers will be subject to the same application process and approval criteria as specified by UK Biobank. For more details on the access procedure, see the UK Biobank website: http://www.ukbiobank.ac.uk/register-apply.

## Declarations:

### Ethnics Approval:

The UK Biobank study was approved by the National Health and Social Care Information Management Board and the North West Multicentre Research Ethics Committee (11/NW/0382), and the Institutional Review Board of Tulane University (2018–1872).

### Transparency Statement:

LQ affirms that the manuscript is an honest, accurate, and transparent account of the study being reported; that no important aspects of the study have been omitted, and that any discrepancies from the study as planned have been explained.
