# Supplementary material for: Joint Physical-Psychosocial Frailty and Risks of All-Cause and Cause-Specific Premature Mortality
Source: J Gen Intern Med. 2025 Jan 22;40(10):2207–17. doi: 10.1007/s11606-024-09335-z (PMC12343393; doi:10.1007/s11606-024-09335-z)
Supplement: Supplementary file 1 — Supplementary file1 (DOCX 5.02 MB) [file 11606_2024_9335_MOESM1_ESM.docx]

**Supplemental Online Content**

Supplement Figure 1. Flow chart.

Supplement Figure 2. Cumulative hazard curves for the probability of all-cause premature mortality and causes-specific premature mortality.

Supplement Figure 3. Dose-response associations of physical-psychosocial frailty index with risk of premature mortality via multivariable model.

Supplement Figure 4. Hazard ratios and 95% confidence intervals for association of physical-psychosocial frailty indicators with outcome of premature mortality via multivariable model.

Supplement Figure 5. Association of physical-psychosocial frailty indicators with risk of all-cause premature mortality stratified by body mass index (BMI) via multivariable model.

Supplement Table 1. Physical-psychosocial frailty index criteria.

Supplement Table 2. Assessment of healthy diet score in the UK Biobank.

Supplement Table 3. The numbers and percentages of participants with missing covariates.

Supplement Table 4. Association of physical-psychosocial frailty with risk of cancer premature mortality stratified by potential risk factors via multivariable model.

Supplement Table 5. Association of physical-psychosocial frailty with risk of CVD premature mortality stratified by potential risk factors via multivariable model.

Supplement Table 6. Association of physical-psychosocial frailty with risk of other premature mortality stratified by potential risk factors via multivariable model.

Supplement Table 7. Interactions between the frailty index components and other covariates.

Supplement Table 8. Hazard ratios and 95% confidence intervals obtained from multivariable model for association of physical-psychosocial frailty index with outcome of premature mortality after removing the participants who were dead within 2 years.

Supplement Table 9. Hazard ratios and 95% confidence intervals obtained from multivariable model for association of physical-psychosocial frailty index with outcome of premature mortality after removing participants with missing data.

Supplement Table 10. Hazard ratios and 95% confidence intervals obtained from multivariable model for association of physical-psychosocial frailty index with outcome of premature mortality with all missing covariate data imputed using multiple imputation.


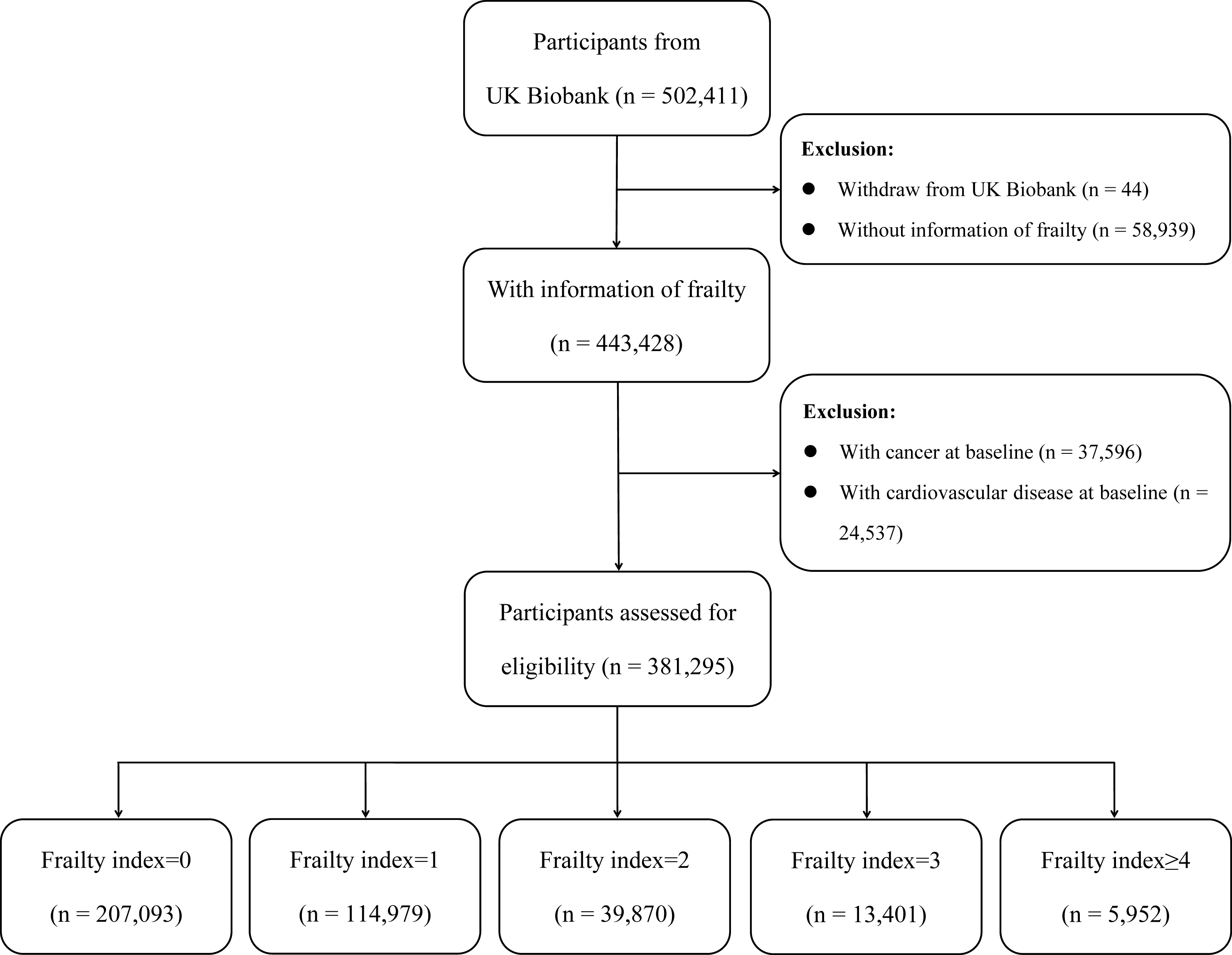


Supplement Figure 1. Flow chart.


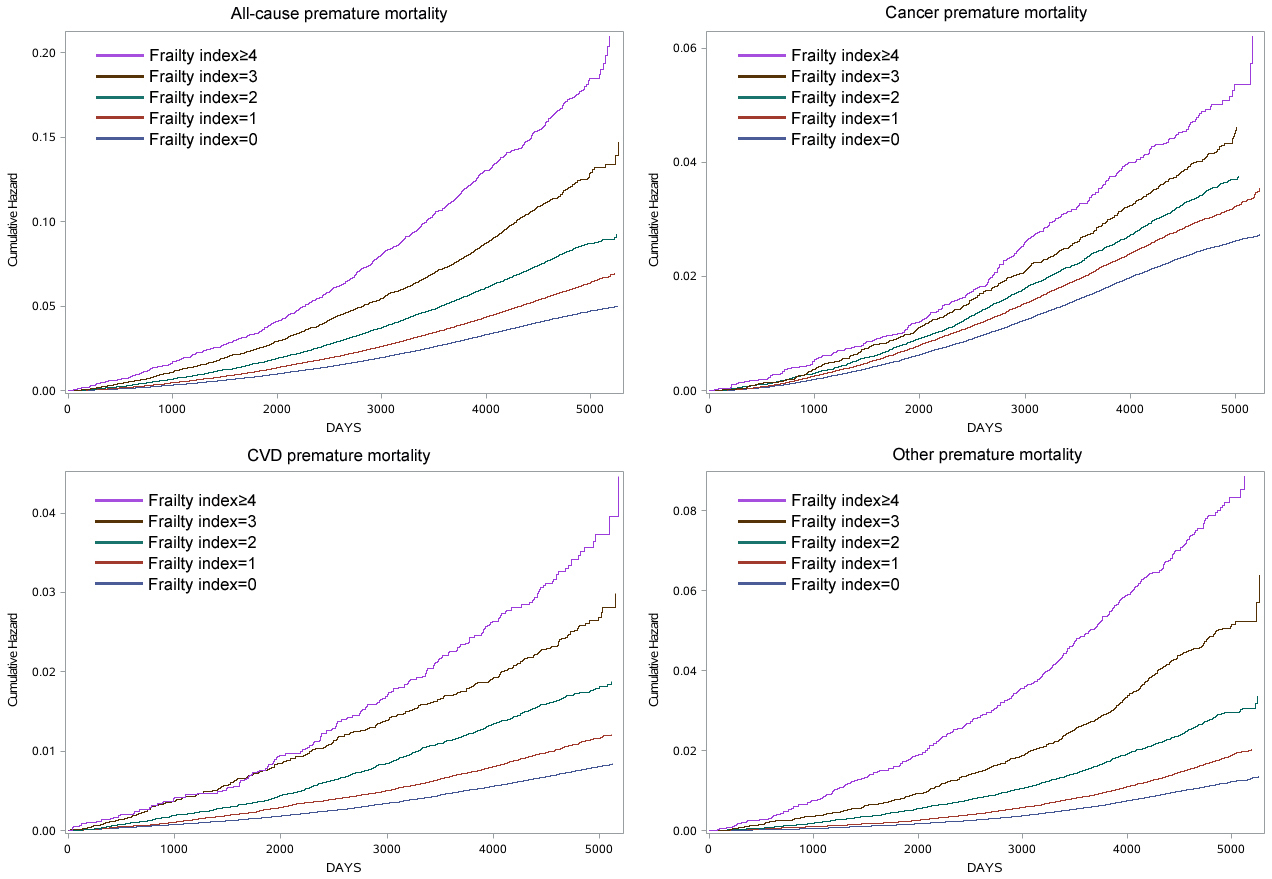


Supplement Figure 2. Cumulative hazard curves for the probability of all-cause premature mortality and causes-specific premature mortality.

CVD: cardiovascular disease.


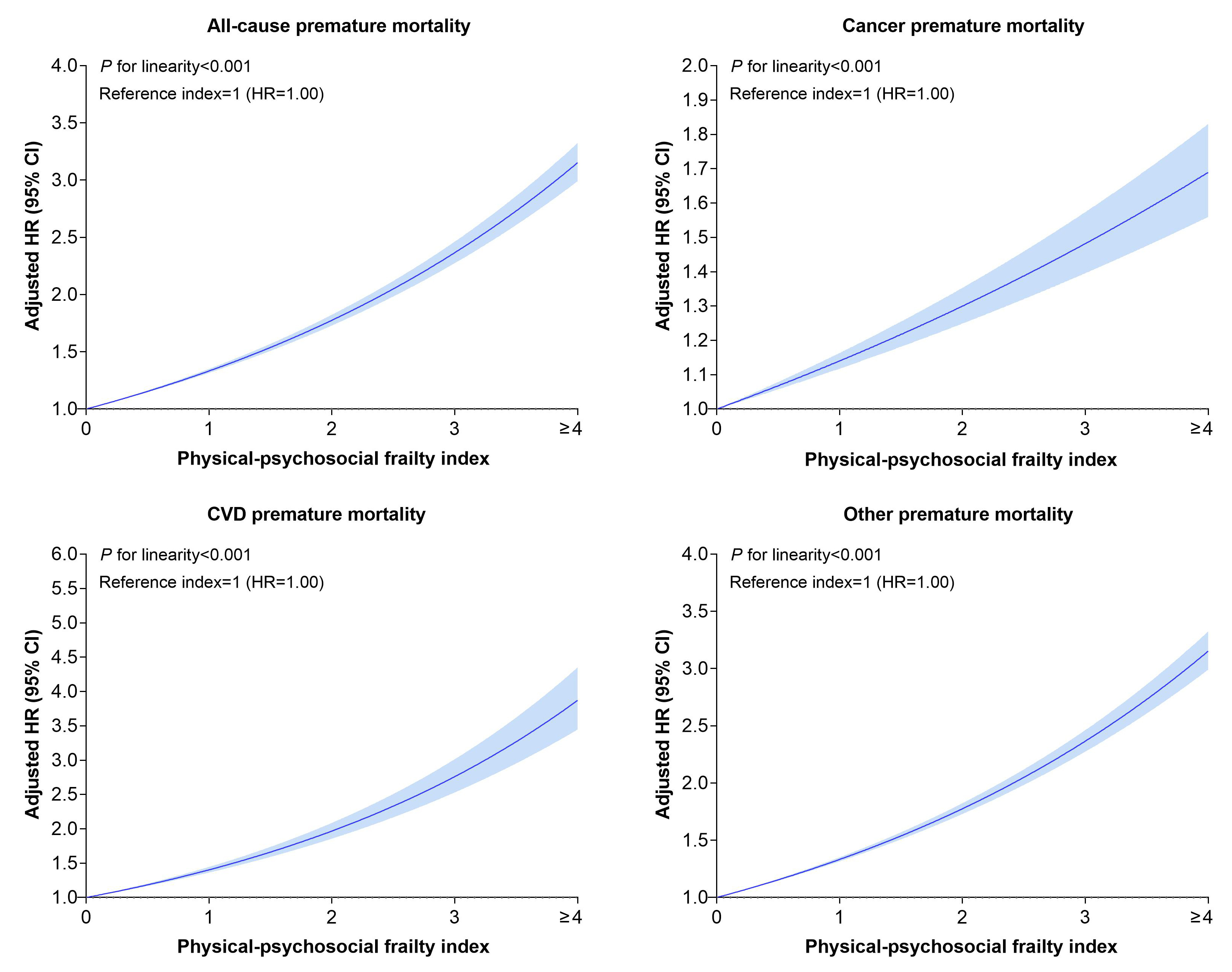


Supplement Figure 3. Dose-response associations of physical-psychosocial frailty index with risk of premature mortality via multivariable model.

CVD: cardiovascular disease.

Multivariable model: adjusted for age, sex, ethnic background, Townsend deprivation index, education years, body mass index, smoking status, alcohol intake, healthy diet score, hypertension, high cholesterol and diabetes.


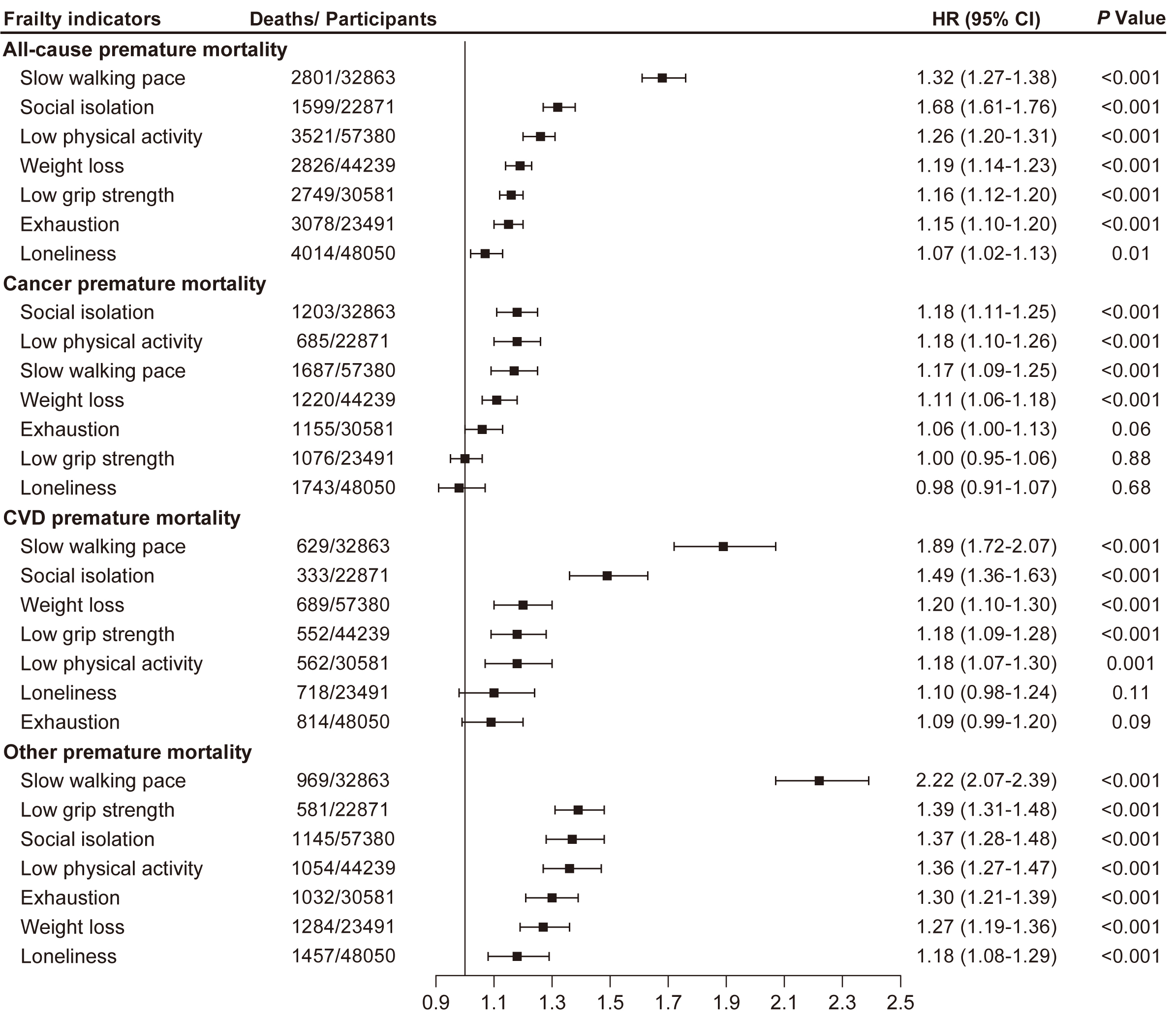
Supplement Figure 4. Hazard ratios and 95% confidence intervals for association of physical-psychosocial frailty indicators with outcome of premature mortality via multivariable model.

CVD: cardiovascular disease.

Multivariable model: adjusted for age, sex, ethnic background, Townsend deprivation index, education years, body mass index, smoking status, alcohol intake, healthy diet score, hypertension, high cholesterol and diabetes. Social isolation, loneliness, weight loss, exhaustion, physical activity, walking pace and grip strength were mutually adjusted for each other.


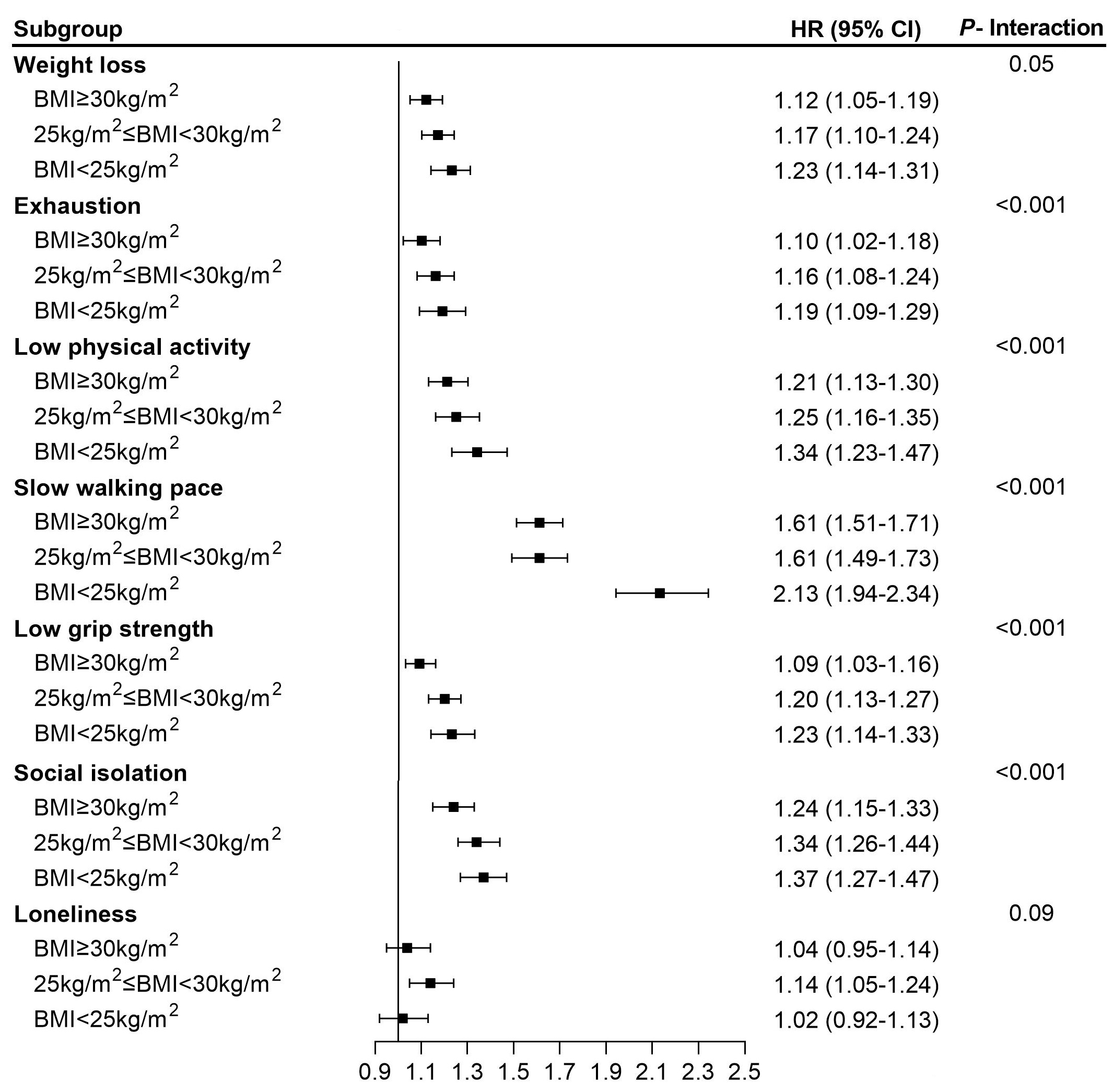


Supplement Figure 5. Association of physical-psychosocial frailty indicators with risk of all-cause premature mortality stratified by body mass index (BMI) via multivariable model.

Multivariable model: adjusted for age, sex, ethnic background, Townsend deprivation index, education years, body mass index, smoking status, alcohol intake, healthy diet score, hypertension, high cholesterol and diabetes. Social isolation, loneliness, weight loss, exhaustion, physical activity, walking pace and grip strength were mutually adjusted for each other.

| **Exposures** | **UK Biobank field ID** | **ACE touchscreen questions** | **Index** | **Responses** |
| --- | --- | --- | --- | --- |
| Weight loss | 2306 | Compared with one year ago, has your weight changed? | 0 | - Other |
|  |  |  | 1 | - Lost weight |
| Exhaustion | 2080 | Over the past two weeks, how often have you felt tired or had little energy? | 0 | - Other |
|  |  |  | 1 | - More than half the days or nearly every day |
| Physical activity | 6164 | In the last 4 weeks did you spend any time doing the following? (You can select more than one answer) | 0 | - Medium or heavy activity |
|  |  |  | 1 | - No activity |
|  | 1011 | How many times in the last 4 weeks did you do light DIY? | 0 | - Light activity more than once per week |
|  |  |  | 1 | - Light activity with a frequency of once per week or less |
| Walking pace | 924 | How would you describe your usual walking pace? | 0 | - Other |
|  |  |  | 1 | - Slow |
| Grip strength | 31 | Sex of participant. | 0 | - Maximal grip strength of left and right hands>cut-off value ^1^ |
|  | 46 | Left grip strength. |  |  |
|  | 47 | Right grip strength. |  |  |
|  | 21001 | BMI value here is constructed from height and weight measured during the initial Assessment Centre visit. | 1 | - Maximal grip strength of left and right hands≤cut-off value ^1^ |
| Social isolation | 709 | Including yourself, how many people are living together in your household? | 0 | - Not living alone. |
|  |  |  | 1 | - Living alone |
|  | 1031 | How often do you visit friends or family or have them visit you? | 0 | - Almost daily - 2-4 times a week - About once a week - About once a month |
|  |  |  | 1 | - Once every few months - Never or almost never - No friends/family outside household |
|  | 6160 | Which of the following (sports club or gym, pub or social club, religious group, adult education class, other group activity) do you attend once a week or more often? | 0 | - Sports club or gym - Pub or social club - Religious group - Adult education class - Other group activity |
|  |  |  | 1 | - None of the above |
| Loneliness | 2020 | Do you often feel lonely? | 0 | - No |
|  |  |  | 1 | - Yes |
|  | 2110 | How often are you able to confide in someone close to you? | 0 | - Almost daily - 2-4 times a week - About once a week - About once a month |
|  |  |  | 1 | - Once every few months - Never or almost never |

Supplement Table 1. Physical-psychosocial frailty index criteria.

Reference

1. L. P. Fried, C. M. Tangen, J. Walston, et al. Frailty in older adults: evidence for a phenotype. J Gerontol A Biol Sci Med Sci 2001;56(3):M146-56. doi: 10.1093/gerona/56.3.m146 pmid:112531562001-03-01].

Supplement Table 2. Assessment of healthy diet score in the UK Biobank.

| **Diet** | **UK Biobank field ID** | **Description** | **Healthy diet score** |
| --- | --- | --- | --- |
| Vegetable | 1289 | Cooked vegetable intake | 1 for ≥4 tablespoons/day  0 for <4 tablespoons/day |
|  | 1299 | Salad/raw vegetable intake |  |
| Fruit | 1309 | Fresh fruit intake | 1 for ≥3 pieces/day  0 for <3 pieces/day |
|  | 1319 | Dried fruit intake |  |
| Fish | 1329 | Oily fish intake | 1 for ≥2 times/week  0 for <2 times/week |
|  | 1339 | Non-oily fish intake |  |
| Processed meat | 1349 | Processed meat intake | 1 for <2 times/week  0 for ≥2 times/week |
| Unprocessed red meat | 1369 | Beef intake | 1 for <2 times/week  0 for ≥2 times/week |
|  | 1379 | Lamb/mutton intake |  |
|  | 1389 | Pork intake |  |

Supplement Table 3. The numbers and percentages of participants with missing covariates.

| **Variable** | **N** | **%** |
| --- | --- | --- |
| Healthy diet score | 10374 | 2.72 |
| Education | 2400 | 0.63 |
| High cholesterol | 1890 | 0.50 |
| Ethnic background | 1012 | 0.27 |
| Smoking status | 938 | 0.25 |
| Townsend deprivation index | 481 | 0.13 |
| Alcohol intake | 153 | 0.04 |
| Diabetes | 45 | 0.01 |

Supplement Table 4. Association of physical-psychosocial frailty with risk of cancer premature mortality stratified by potential risk factors via multivariable model.

| **Subgroup** | **Physical-psychosocial frailty index** | | | | | ***P-*trend** | ***P*-interaction** |
| --- | --- | --- | --- | --- | --- | --- | --- |
|  | **0** | **1** | **2** | **3** | **≥4** |  |  |
| Age (years) |  |  |  |  |  |  | 0.550 |
| <60 | 1.00 (reference) | 1.18 (1.10-1.28) | 1.32 (1.19-1.47) | 1.41 (1.20-1.65) | 1.70 (1.39-2.09) | <0.001 |  |
| ≥60 | 1.00 (reference) | 1.16 (1.10-1.22) | 1.23 (1.14-1.33) | 1.39 (1.24-1.56) | 1.51 (1.29-1.77) | <0.001 |  |
| Sex |  |  |  |  |  |  | 0.845 |
| Female | 1.00 (reference) | 1.15 (1.08-1.23) | 1.23 (1.13-1.35) | 1.43 (1.26-1.62) | 1.66 (1.40-1.96) | <0.001 |  |
| Male | 1.00 (reference) | 1.18 (1.11-1.25) | 1.27 (1.16-1.38) | 1.34 (1.17-1.53) | 1.48 (1.23-1.78) | <0.001 |  |
| Ethnic background |  |  |  |  |  |  | 0.243 |
| Non-white | 1.00 (reference) | 1.28 (1.07-1.52) | 1.33 (1.06-1.66) | 1.06 (0.74-1.50) | 1.47 (0.97-2.23) | 0.035 |  |
| White | 1.00 (reference) | 1.16 (1.10-1.21) | 1.24 (1.17-1.33) | 1.43 (1.30-1.57) | 1.59 (1.39-1.81) | <0.001 |  |
| Townsend deprivation index | |  |  |  |  |  | 0.208 |
| <Median | 1.00 (reference) | 1.14 (1.07-1.21) | 1.24 (1.12-1.36) | 1.31 (1.11-1.54) | 1.24 (0.94-1.64) | <0.001 |  |
| ≥Median | 1.00 (reference) | 1.20 (1.13-1.28) | 1.30 (1.19-1.41) | 1.48 (1.32-1.66) | 1.76 (1.53-2.03) | <0.001 |  |
| Education (years) |  |  |  |  |  |  | 0.755 |
| <10 | 1.00 (reference) | 1.11 (1.01-1.22) | 1.22 (1.08-1.37) | 1.33 (1.14-1.55) | 1.53 (1.27-1.85) | <0.001 |  |
| ≥10 | 1.00 (reference) | 1.18 (1.12-1.24) | 1.26 (1.17-1.35) | 1.39 (1.24-1.57) | 1.51 (1.27-1.79) | <0.001 |  |
| Smoking status |  |  |  |  |  |  | <0.001 |
| Never | 1.00 (reference) | 1.10 (1.03-1.18) | 1.17 (1.05-1.29) | 1.17 (0.99-1.38) | 1.21 (0.94-1.55) | 0.003 |  |
| Previous/Current | 1.00 (reference) | 1.21 (1.14-1.28) | 1.30 (1.20-1.41) | 1.50 (1.34-1.67) | 1.69 (1.46-1.95) | <0.001 |  |
| Alcohol intake (times/week) | |  |  |  |  |  | 0.302 |
| <3 | 1.00 (reference) | 1.13 (1.06-1.20) | 1.20 (1.11-1.31) | 1.31 (1.17-1.47) | 1.50 (1.29-1.74) | <0.001 |  |
| ≥3 | 1.00 (reference) | 1.19 (1.12-1.27) | 1.30 (1.18-1.43) | 1.51 (1.29-1.78) | 1.72 (1.35-2.18) | <0.001 |  |
| Healthy diet score |  |  |  |  |  |  | 0.710 |
| <3 | 1.00 (reference) | 1.17 (1.09-1.26) | 1.23 (1.11-1.37) | 1.44 (1.25-1.66) | 1.59 (1.32-1.92) | <0.001 |  |
| ≥3 | 1.00 (reference) | 1.15 (1.09-1.22) | 1.26 (1.17-1.37) | 1.30 (1.14-1.48) | 1.46 (1.21-1.75) | <0.001 |  |
| High cholesterol |  |  |  |  |  |  | 0.193 |
| No | 1.00 (reference) | 1.15 (1.09-1.21) | 1.29 (1.20-1.38) | 1.40 (1.25-1.56) | 1.59 (1.36-1.86) | <0.001 |  |
| Yes | 1.00 (reference) | 1.21 (1.10-1.34) | 1.13 (0.99-1.29) | 1.34 (1.12-1.59) | 1.40 (1.12-1.75) | <0.001 |  |
| Diabetes |  |  |  |  |  |  | 0.982 |
| No | 1.00 (reference) | 1.16 (1.11-1.22) | 1.25 (1.17-1.34) | 1.38 (1.25-1.53) | 1.55 (1.35-1.78) | <0.001 |  |
| Yes | 1.00 (reference) | 1.19 (0.99-1.43) | 1.21 (0.98-1.50) | 1.40 (1.08-1.81) | 1.60 (1.19-2.15) | 0.004 |  |

Multivariable model: adjusted for age, sex, ethnic background, Townsend deprivation index, education years, body mass index, smoking status, alcohol intake, healthy diet score, hypertension, high cholesterol and diabetes.

Supplement Table 5. Association of physical-psychosocial frailty with risk of CVD premature mortality stratified by potential risk factors via multivariable model.

| **Subgroup** | **Physical-psychosocial frailty index** | | | | | ***P-*trend** | ***P*-interaction** |
| --- | --- | --- | --- | --- | --- | --- | --- |
|  | **0** | **1** | **2** | **3** | **≥4** |  |  |
| Age (years) |  |  |  |  |  |  | 0.017 |
| <60 | 1.00 (reference) | 1.21 (1.05-1.39) | 2.00 (1.70-2.36) | 2.54 (2.05-3.16) | 3.10 (2.40-4.01) | <0.001 |  |
| ≥60 | 1.00 (reference) | 1.40 (1.28-1.54) | 1.86 (1.65-2.09) | 2.48 (2.12-2.90) | 2.92 (2.39-3.58) | <0.001 |  |
| Sex |  |  |  |  |  |  | 0.546 |
| Female | 1.00 (reference) | 1.28 (1.11-1.47) | 1.85 (1.57-2.18) | 2.13 (1.71-2.65) | 3.00 (2.33-3.87) | <0.001 |  |
| Male | 1.00 (reference) | 1.37 (1.25-1.50) | 1.92 (1.71-2.16) | 2.72 (2.33-3.18) | 2.99 (2.44-3.67) | <0.001 |  |
| Ethnic background |  |  |  |  |  |  | 0.220 |
| Non-white | 1.00 (reference) | 1.44 (1.06-1.94) | 2.49 (1.79-3.46) | 2.51 (1.62-3.88) | 2.31 (1.30-4.10) | <0.001 |  |
| White | 1.00 (reference) | 1.33 (1.23-1.44) | 1.84 (1.66-2.03) | 2.52 (2.21-2.87) | 3.07 (2.60-3.63) | <0.001 |  |
| Townsend deprivation index | |  |  |  |  |  | 0.373 |
| <Median | 1.00 (reference) | 1.22 (1.09-1.37) | 1.84 (1.58-2.15) | 2.43 (1.93-3.05) | 3.05 (2.18-4.27) | <0.001 |  |
| ≥Median | 1.00 (reference) | 1.46 (1.31-1.62) | 2.02 (1.79-2.29) | 2.69 (2.30-3.13) | 3.30 (2.75-3.95) | <0.001 |  |
| Education (years) |  |  |  |  |  |  | 0.621 |
| <10 | 1.00 (reference) | 1.46 (1.25-1.71) | 2.01 (1.68-2.40) | 2.47 (1.99-3.06) | 3.17 (2.46-4.08) | <0.001 |  |
| ≥10 | 1.00 (reference) | 1.30 (1.19-1.42) | 1.84 (1.64-2.07) | 2.57 (2.20-3.01) | 2.96 (2.40-3.64) | <0.001 |  |
| Smoking status |  |  |  |  |  |  | 0.463 |
| Never | 1.00 (reference) | 1.25 (1.11-1.40) | 1.65 (1.42-1.93) | 2.38 (1.94-2.93) | 2.70 (2.06-3.55) | <0.001 |  |
| Previous/Current | 1.00 (reference) | 1.41 (1.28-1.56) | 2.07 (1.83-2.34) | 2.56 (2.18-3.00) | 3.15 (2.58-3.83) | <0.001 |  |
| Alcohol intake (times/week) | |  |  |  |  |  | 0.069 |
| <3 | 1.00 (reference) | 1.40 (1.26-1.56) | 1.92 (1.69-2.18) | 2.35 (2.01-2.76) | 2.88 (2.38-3.50) | <0.001 |  |
| ≥3 | 1.00 (reference) | 1.26 (1.13-1.41) | 1.87 (1.61-2.17) | 2.90 (2.35-3.57) | 3.35 (2.50-4.49) | <0.001 |  |
| Healthy diet score |  |  |  |  |  |  | 0.042 |
| <3 | 1.00 (reference) | 1.44 (1.26-1.64) | 2.27 (1.95-2.64) | 2.81 (2.32-3.40) | 3.45 (2.74-4.35) | <0.001 |  |
| ≥3 | 1.00 (reference) | 1.28 (1.16-1.41) | 1.71 (1.50-1.95) | 2.17 (1.81-2.61) | 2.70 (2.12-3.43) | <0.001 |  |
| High cholesterol |  |  |  |  |  |  | 0.312 |
| No | 1.00 (reference) | 1.27 (1.16-1.39) | 1.84 (1.64-2.06) | 2.49 (2.13-2.91) | 2.92 (2.37-3.59) | <0.001 |  |
| Yes | 1.00 (reference) | 1.51 (1.30-1.75) | 2.05 (1.72-2.44) | 2.46 (1.97-3.07) | 3.20 (2.48-4.14) | <0.001 |  |
| Diabetes |  |  |  |  |  |  | 0.598 |
| No | 1.00 (reference) | 1.36 (1.25-1.47) | 1.93 (1.74-2.13) | 2.51 (2.18-2.89) | 2.93 (2.43-3.53) | <0.001 |  |
| Yes | 1.00 (reference) | 1.12 (0.87-1.44) | 1.61 (1.24-2.10) | 2.16 (1.60-2.93) | 2.88 (2.08-3.99) | <0.001 |  |

Multivariable model: adjusted for age, sex, ethnic background, Townsend deprivation index, education years, body mass index, smoking status, alcohol intake, healthy diet score, hypertension, high cholesterol and diabetes.

Supplement Table 6. Association of physical-psychosocial frailty with risk of other premature mortality stratified by potential risk factors via multivariable model.

| **Subgroup** | **Physical-psychosocial frailty index** | | | | | ***P-*trend** | ***P*-interaction** |
| --- | --- | --- | --- | --- | --- | --- | --- |
|  | **0** | **1** | **2** | **3** | **≥4** |  |  |
| Age (years) |  |  |  |  |  |  | <0.001 |
| <60 | 1.00 (reference) | 1.51 (1.34-1.69) | 2.25 (1.96-2.58) | 3.83 (3.25-4.51) | 6.36 (5.33-7.59) | <0.001 |  |
| ≥60 | 1.00 (reference) | 1.39 (1.29-1.50) | 2.09 (1.90-2.29) | 3.23 (2.87-3.64) | 4.27 (3.69-4.94) | <0.001 |  |
| Sex |  |  |  |  |  |  | 0.534 |
| Female | 1.00 (reference) | 1.39 (1.26-1.54) | 2.05 (1.81-2.32) | 3.44 (2.97-3.99) | 5.30 (4.49-6.25) | <0.001 |  |
| Male | 1.00 (reference) | 1.44 (1.33-1.56) | 2.16 (1.95-2.39) | 3.31 (2.91-3.75) | 4.66 (4.00-5.42) | <0.001 |  |
| Ethnic background |  |  |  |  |  |  | 0.553 |
| Non-white | 1.00 (reference) | 1.25 (0.97-1.60) | 1.71 (1.28-2.29) | 2.86 (2.04-3.99) | 4.56 (3.16-6.58) | <0.001 |  |
| White | 1.00 (reference) | 1.44 (1.35-1.53) | 2.16 (1.99-2.34) | 3.43 (3.10-3.79) | 5.01 (4.46-5.64) | <0.001 |  |
| Townsend deprivation index | |  |  |  |  |  | 0.564 |
| <Median | 1.00 (reference) | 1.38 (1.26-1.51) | 2.22 (1.96-2.50) | 3.75 (3.18-4.41) | 5.56 (4.44-6.95) | <0.001 |  |
| ≥Median | 1.00 (reference) | 1.49 (1.37-1.63) | 2.17 (1.96-2.40) | 3.47 (3.08-3.90) | 5.27 (4.63-6.00) | <0.001 |  |
| Education (years) |  |  |  |  |  |  | 0.042 |
| <10 | 1.00 (reference) | 1.38 (1.21-1.56) | 1.85 (1.60-2.14) | 3.34 (2.86-3.91) | 4.45 (3.71-5.33) | <0.001 |  |
| ≥10 | 1.00 (reference) | 1.44 (1.34-1.55) | 2.25 (2.05-2.47) | 3.40 (3.00-3.84) | 5.39 (4.67-6.24) | <0.001 |  |
| Smoking status |  |  |  |  |  |  | 0.981 |
| Never | 1.00 (reference) | 1.42 (1.29-1.57) | 2.07 (1.83-2.34) | 3.34 (2.85-3.91) | 5.03 (4.16-6.07) | <0.001 |  |
| Previous/Current | 1.00 (reference) | 1.41 (1.30-1.54) | 2.14 (1.93-2.36) | 3.38 (3.00-3.82) | 4.89 (4.25-5.62) | <0.001 |  |
| Alcohol intake (times/week) | |  |  |  |  |  | 0.002 |
| <3 | 1.00 (reference) | 1.37 (1.26-1.50) | 1.93 (1.75-2.14) | 3.16 (2.81-3.56) | 4.35 (3.79-4.99) | <0.001 |  |
| ≥3 | 1.00 (reference) | 1.47 (1.34-1.61) | 2.36 (2.10-2.66) | 3.75 (3.19-4.41) | 6.64 (5.48-8.04) | <0.001 |  |
| Healthy diet score |  |  |  |  |  |  | 0.654 |
| <3 | 1.00 (reference) | 1.40 (1.26-1.56) | 2.11 (1.85-2.40) | 3.61 (3.12-4.19) | 5.00 (4.21-5.93) | <0.001 |  |
| ≥3 | 1.00 (reference) | 1.43 (1.32-1.55) | 2.11 (1.91-2.34) | 3.21 (2.81-3.67) | 4.85 (4.13-5.69) | <0.001 |  |
| High cholesterol |  |  |  |  |  |  | 0.003 |
| No | 1.00 (reference) | 1.43 (1.33-1.54) | 2.23 (2.04-2.44) | 3.56 (3.18-3.99) | 5.63 (4.92-6.44) | <0.001 |  |
| Yes | 1.00 (reference) | 1.36 (1.20-1.55) | 1.71 (1.46-2.00) | 2.80 (2.34-3.35) | 3.79 (3.09-4.65) | <0.001 |  |
| Diabetes |  |  |  |  |  |  | 0.001 |
| No | 1.00 (reference) | 1.43 (1.34-1.53) | 2.21 (2.04-2.40) | 3.47 (3.12-3.85) | 5.56 (4.92-6.29) | <0.001 |  |
| Yes | 1.00 (reference) | 1.27 (1.04-1.55) | 1.50 (1.20-1.87) | 2.64 (2.08-3.35) | 3.09 (2.38-4.02) | <0.001 |  |

Multivariable model: adjusted for age, sex, ethnic background, Townsend deprivation index, education years, body mass index, smoking status, alcohol intake, healthy diet score, hypertension, high cholesterol and diabetes.

Supplement Table 7. Interactions between the frailty index components and other covariates.

| **Subgroup** | ***P*-interaction** | | | | | | |
| --- | --- | --- | --- | --- | --- | --- | --- |
|  | **Weight loss** | **Exhaustion** | **Physical activity** | **Walking pace** | **Grip strength** | **Social isolation** | **Loneliness** |
| Age | 0.160 | 0.067 | 0.006 | <0.001 | <0.001 | 0.002 | 0.424 |
| Sex | 0.289 | 0.623 | 0.049 | 0.246 | 0.004 | 0.025 | 0.559 |
| Ethnic background | 0.531 | 0.605 | 0.081 | 0.011 | 0.693 | 0.282 | 0.569 |
| Townsend deprivation index | 0.322 | 0.700 | 0.123 | 0.771 | 0.703 | 0.019 | 0.079 |
| Education | 0.947 | 0.646 | 0.936 | 0.698 | 0.018 | 0.917 | 0.234 |
| Body Mass Index | 0.004 | 0.004 | <0.001 | <0.001 | <0.001 | 0.004 | 0.353 |
| Smoking status | 0.250 | 0.554 | 0.140 | 0.669 | 0.372 | 0.018 | 0.291 |
| Alcohol intake | 0.823 | 0.106 | <0.001 | 0.001 | 0.454 | 0.002 | 0.818 |
| Healthy diet score | 0.466 | 0.541 | 0.389 | 0.557 | 0.084 | 0.014 | 0.323 |
| High cholesterol | 0.155 | 0.072 | 0.201 | 0.061 | 0.005 | 0.906 | 0.519 |
| Diabetes | 0.030 | 0.648 | 0.450 | 0.658 | 0.532 | 0.898 | 0.857 |

Supplement Table 8. Hazard ratios and 95% confidence intervals obtained from multivariable model for association of physical-psychosocial frailty index with outcome of premature mortality after removing the participants who were dead within 2 years.

| **Outcomes** | **Physical-psychosocial frailty index** | | | | | **Per 1 index increase** | ***P*-trend** |
| --- | --- | --- | --- | --- | --- | --- | --- |
|  | **0** | **1** | **2** | **3** | **≥4** |  |  |
| All-cause premature mortality | 1.00 (Reference) | 1.25 (1.21-1.29) | 1.55 (1.48-1.62) | 2.04 (1.92-2.16) | 2.60 (2.41-2.80) | 1.25 (1.24-1.27) | <0.001 |
| Cancer premature mortality | 1.00 (Reference) | 1.15 (1.10-1.20) | 1.20 (1.12-1.28) | 1.32 (1.20-1.45) | 1.41 (1.24-1.60) | 1.09 (1.07-1.11) | <0.001 |
| CVD premature mortality | 1.00 (Reference) | 1.34 (1.24-1.45) | 1.86 (1.68-2.05) | 2.31 (2.02-2.64) | 2.79 (2.36-3.29) | 1.30 (1.26-1.34) | <0.001 |
| Other premature mortality | 1.00 (Reference) | 1.39 (1.30-1.48) | 2.03 (1.88-2.20) | 3.16 (2.86-3.48) | 4.51 (4.02-5.06) | 1.43 (1.40-1.46) | <0.001 |

CVD: cardiovascular disease.

Multivariable model: adjusted for age, sex, ethnic background, Townsend deprivation index, education years, body mass index, smoking status, alcohol intake, healthy diet score, hypertension, high cholesterol and diabetes.

Supplement Table 9. Hazard ratios and 95% confidence intervals obtained from multivariable model for association of physical-psychosocial frailty index with outcome of premature mortality after removing participants with missing data.

| **Outcomes** | **Physical-psychosocial frailty index** | | | | | **Per 1 index increase** | ***P*-trend** |
| --- | --- | --- | --- | --- | --- | --- | --- |
|  | **0** | **1** | **2** | **3** | **≥4** |  |  |
| All-cause premature mortality | 1.00 (Reference) | 1.25 (1.21-1.30) | 1.57 (1.50-1.64) | 2.06 (1.94-2.19) | 2.64 (2.44-2.85) | 1.26 (1.24-1.28) | <0.001 |
| Cancer premature mortality | 1.00 (Reference) | 1.15 (1.10-1.20) | 1.22 (1.15-1.31) | 1.32 (1.19-1.45) | 1.40 (1.22-1.60) | 1.10 (1.07-1.12) | <0.001 |
| CVD premature mortality | 1.00 (Reference) | 1.32 (1.22-1.43) | 1.87 (1.69-2.06) | 2.30 (2.01-2.63) | 2.77 (2.34-3.29) | 1.30 (1.26-1.34) | <0.001 |
| Other premature mortality | 1.00 (Reference) | 1.42 (1.33-1.52) | 2.06 (1.90-2.23) | 3.29 (2.98-3.64) | 4.74 (4.21-5.34) | 1.45 (1.42-1.48) | <0.001 |

CVD: cardiovascular disease.

Multivariable model: adjusted for age, sex, ethnic background, Townsend deprivation index, education years, body mass index, smoking status, alcohol intake, healthy diet score, hypertension, high cholesterol and diabetes.

Supplement Table 10. Hazard ratios and 95% confidence intervals obtained from multivariable model for association of physical-psychosocial frailty index with outcome of premature mortality with all missing covariate data imputed using multiple imputation.

| **Outcomes** | **Physical-psychosocial frailty index** | | | | | **Per 1 index increase** | ***P*-trend** |
| --- | --- | --- | --- | --- | --- | --- | --- |
|  | **0** | **1** | **2** | **3** | **≥4** |  |  |
| All-cause premature mortality | 1.00 (Reference) | 1.26 (1.22-1.30) | 1.57 (1.51-1.64) | 2.09 (1.97-2.21) | 2.71 (2.52-2.91) | 1.26 (1.25-1.28) | <0.001 |
| Cancer premature mortality | 1.00 (Reference) | 1.15 (1.10-1.20) | 1.22 (1.14-1.29) | 1.32 (1.21-1.45) | 1.46 (1.29-1.65) | 1.10 (1.08-1.12) | <0.001 |
| CVD premature mortality | 1.00 (Reference) | 1.32 (1.23-1.43) | 1.85 (1.68-2.03) | 2.37 (2.09-2.69) | 2.80 (2.39-3.27) | 1.30 (1.26-1.34) | <0.001 |
| Other premature mortality | 1.00 (Reference) | 1.41 (1.33-1.50) | 2.08 (1.93-2.25) | 3.29 (2.99-3.61) | 4.76 (4.26-5.32) | 1.45 (1.42-1.48) | <0.001 |

CVD: cardiovascular disease.

Multivariable model: adjusted for age, sex, ethnic background, Townsend deprivation index, education years, body mass index, smoking status, alcohol intake, healthy diet score, hypertension, high cholesterol and diabetes.
